# Function and Structural Organization of the Replication Protein of *Bamboo mosaic virus*

**DOI:** 10.3389/fmicb.2017.00522

**Published:** 2017-03-28

**Authors:** Menghsiao Meng, Cheng-Cheng Lee

**Affiliations:** Graduate Institute of Biotechnology, National Chung Hsing UniversityTaichung, Taiwan

**Keywords:** *Bamboo mosaic virus*, *Potexvirus*, RNA-dependent RNA polymerase, mRNA capping, virus-host interaction, positive-strand RNA virus, guanylyltransferase

## Abstract

The genus *Potexvirus* is one of the eight genera belonging to the family *Alphaflexiviridae* according to the Virus Taxonomy 2015 released by International Committee on Taxonomy of Viruses (www.ictvonline.org/index.asp). Currently, the genus contains 35 known species including many agricultural important viruses, e.g., *Potato virus X* (PVX). Members of this genus are characterized by flexuous, filamentous virions of 13 nm in diameter and 470–580 nm in length. A potexvirus has a monopartite positive-strand RNA genome, encoding five open-reading frames (ORFs), with a cap structure at the 5′ end and a poly(A) tail at the 3′ end. Besides PVX, *Bamboo mosaic virus* (BaMV) is another potexvirus that has received intensive attention due to the wealth of knowledge on the molecular biology of the virus. In this review, we discuss the enzymatic activities associated with each of the functional domains of the BaMV replication protein, a 155-kDa polypeptide encoded by ORF1. The unique cap formation mechanism, which may be conserved across the alphavirus superfamily, is particularly addressed. The recently identified interactions between the replication protein and the plant host factors are also described.

## BaMv Genome

**FIGURE 1:**
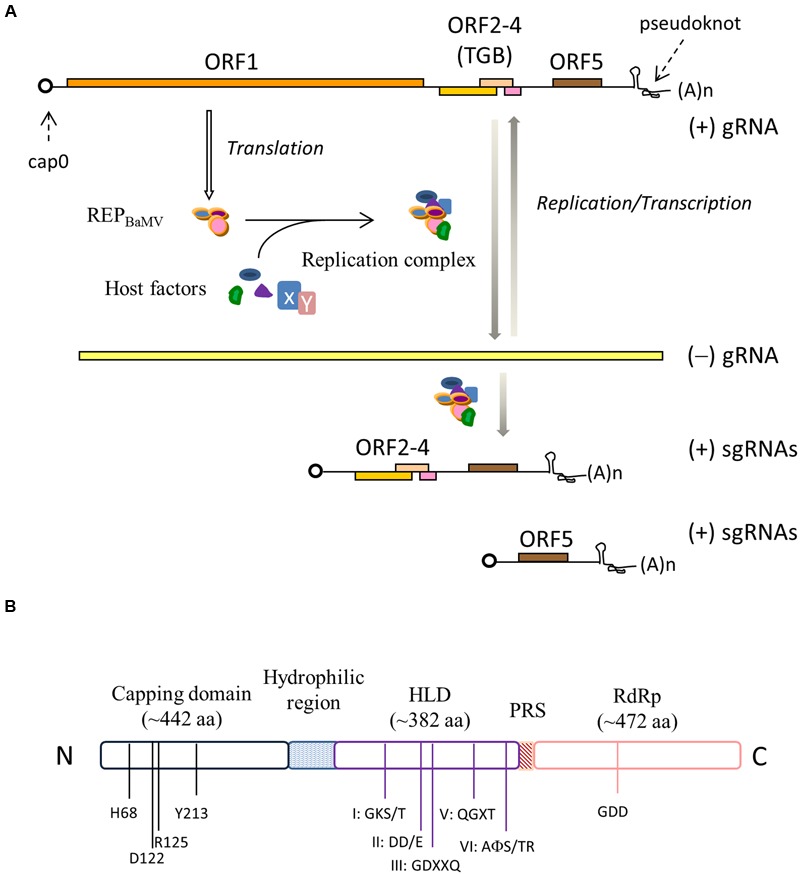
**(A)** The genome organization of *Bamboo mosaic virus* (BaMV). REP_BaMV_, the translation product of ORF1, associates with host factors to form the viral replication complex. Different subsets of host factors may be recruited to perform negative- and positive-strand replication and transcription. **(B)** Functional domains in RFP_BaMV_. The conserved residues in the N-terminal capping enzyme domain and the featured motifs suggestive of the helicase-like domain (HLD) and the C-terminal RdRp domain are indicated. The domains are separated by a disordered hydrophilic region and a proline rich segment (PRS).

*Bamboo mosaic virus* (BaMV) primarily infects members of the *Bambusoideae* in nature; nonetheless, it also replicates in *Nicotiana benthamiana*, which thereby has been used as the surrogate in laboratories. The RNA genome of BaMV contains 6366 nucleotides (nts) plus a 5′ m^7^GpppG (cap0) structure and a 3′ poly(A) tail (**Figure 1A**). It is functionally organized into a 94-nt 5′ untranslated region (UTR), five ORFs, and a 142-nt 3′ UTR (Lin et al., 1994). Two major subgenomic RNAs, co-terminal with the viral 3′ UTR, would be produced once the virus starts to replicate in host cells. The first ORF encodes a 155-kDa non-structural protein (REP_BaMV_) that has been thought to be essential for replication/transcription of the viral genome and the formation of the 5′ cap based on the presence of signature motifs of Sindbis virus-like methyltransferase (Rozanov et al., 1992), helicase (Habili and Symons, 1989), and RNA polymerase (Koonin and Dolja, 1993). As many positive strand RNA viruses, BaMV must encode its own enzymes for replication/transcription and 5′ cap formation because it replicates only in the cytoplasm. ORF2, 3 and 4 are overlapped, often referred to as the triple gene block (TGB), and their translated proteins, TGBp1, TGBp2, and TGBp3, respectively, are indispensable for BaMV movement in plants (Lin et al., 2004, 2006). In-depth discussions about the functions of each of the TGB proteins of PVX in the intracellular trafficking and intercellular transport can be referred in a couple of recent reviews (Verchot-Lubicz et al., 2010; Park et al., 2014). ORF5 encodes the viral coat protein (CP) that is the only structural protein required for the assembly of BaMV virions. CP also exerts a critical function in the accumulation of BaMV RNAs in protoplasts (Lee et al., 2011). It is unclear whether CP protects BaMV RNAs from being destroyed by the host defense mechanisms or if it actually participates in the viral replication process. In addition, CP of potexvirus was reported to play a role in the virus movement. For instance, *White clover mosaic virus* needs CP to spread efficiently in plants (Forster et al., 1992), and PVX is defective in cell-to-cell movement if it carries a C-terminally truncated CP (Fedorkin et al., 2001). Occasionally, an 836-nt satellite RNA (satBaMV) is found in association with BaMV in nature (Lin and Hsu, 1994). satBaMV contains one ORF that encodes a 20-kDa polypeptide (P20). P20 is not necessary for the replication of satBaMV; nonetheless, the accumulation rate of satBaMV in systemic leaves decreases in the absence of P20 (Lin et al., 1996).

## Domain Organization oF Rep_BaMv_

There are apparently three functional domains in REP_BaMV_ (**Figure 1B**), separated by a disordered hydrophilic region, from approximately amino acid residues 406–520, and a proline-rich segment (PRS), residues 895–910, according to a secondary structure prediction using the PHD algorithm (Rost et al., 1994). The N-terminal one-third of REP_BaMV_ shares a few dispersedly conserved residues with the putative Sindbis-like methyltransferase domains (Rozanov et al., 1992) of a variety of plant and animal alphavirus-like viruses such as *Brome mosaic virus* and *Semliki Forest virus* (Li et al., 2001a). Sequence comparison also revealed that the central domain contains several NTP-binding motifs of RNA helicase superfamily 1 (SF1) (Kadaré and Haenni, 1997) and the C-terminal domain contains featured motifs of RNA polymerases, e.g., the catalytic GDD motif (Koonin and Dolja, 1993). Since REP_BaMV_ is barely discernible in BaMV-infected *N. benthamiana*, the enzymatic activity associated with each of the domains has been investigated using the domains expressed in heterologous hosts such as *Escherichia coli* and *Saccharomyces cerevisiae*.

## Capping Enzyme Domain

The enzymatic activity of the N-terminal 442 amino acids of REP_BaMV_ was successfully characterized by using the domain expressed in *S. cerevisiae* (Li et al., 2001a). The recombinant domain, strongly associated with the yeast membrane, could be radiolabeled by [α-^32^P]GTP if *S*-adenosylmethionine (AdoMet) was provided in the reaction buffer. Alternatively, it could be radiolabeled by Ado[*methyl*-^3^H]Met when GTP was present. The radiolabeled moiety covalently linked to the domain was subsequently determined to be m^7^GMP. This [m^7^GMP-enzyme] adduct was thought to represent an intermediate in the pathway to form the 5′ cap. In other words, this viral domain could be a guanylyltransferase (mRNA capping enzyme) except that it is covalently modified by m^7^GMP rather than GMP. In addition, this N-terminal domain of REP_BaMV_ was found capable of catalyzing a methyl transfer reaction from AdoMet to GTP, leading to the formation of m^7^GTP, consistent with the prediction of its function as a methyltransferase. This viral domain was therefore proposed to possess an AdoMet-dependent guanylyltransferase activity, by which the methyl group of AdoMet is transferred to GTP, leading to m^7^GTP formation, and then the m^7^GMP moiety of m^7^GTP was transferred to an active-site residue to form the covalent [m^7^GMP-enzyme] intermediate. Analogous reactions have been observed also in other members of alphavirus-like superfamily including alphavirus (Ahola and Kääriäinen, 1995), *Brome mosaic virus* (Ahola and Ahlquist, 1999), *Semliki Forest virus* (Ahola et al., 1997), *Hepatitis E virus* (Magden et al., 2001) and *Tobacco mosaic virus* (TMV) (Merits et al., 1999), suggesting that this unique mRNA capping process is conserved throughout diverse members within the superfamily in spite of the fact that only limited amino acid identities (e.g., H68, D122, R125, and Y213 in REP_BaMV_) are conserved.

Site-directed mutagenesis indicated that H68, D122, R125, and Y213 are essential for the BaMV capping domain to form the covalent [m^7^GMP-enzyme] intermediate (Huang et al., 2004). Alanine substitution for each of the conserved residues, except H68, also disabled the domain to produce m^7^GTP (Huang et al., 2004). Intriguingly, H68A mutant increased m^7^GTP production by a factor of ∼10, implying a special role of H68 in the pathway to form the covalent [m^7^GMP-enzyme] intermediate. The H68A mutant was thus treated as the pseudo wild type to investigate the aromatic residues important for the formation of m^7^GTP (Hu et al., 2011). A number of aromatic residues, including Y126, F144, F161, Y192, Y203, Y213, and W222, were found critical for AdoMet recognition. Alanine substitution for these residues, except Y213, also reduced the binding affinity to GTP. Probably, the BaMV capping domain binds AdoMet and GTP in close proximity and many of these aromatic residues participate in the binding of the two substrates simultaneously. It is noteworthy that all the indicated aromatic residues are well conserved among the capping domains of potexviruses. The primary function of Y213 is to bind AdoMet. The inability to substitute phenylalanine for Y213 suggests that the hydroxyl group on Y213 provides an essential hydrogen bond to AdoMet. Presumably, Y231 locks AdoMet in a correct spatial position so that the methyl group from the electrophilic methylsulfonium of AdoMet can be transferred to the N7 of GTP.

Peptide mapping using alkaline hydroxylamine, which specifically cleaves the asparaginyl-glycyl bond (Bornstein and Balian, 1977), indicated that the m^7^GMP-linking residue of the BaMV capping domain is located within the region of residues 44–76 (Lin et al., 2012). The covalent [m^7^GMP-enzyme] intermediate was sensitive to 0.1 N HCl but tolerant of 0.1 N NaOH (Lin et al., 2012), suggesting that the link connecting the domain and m^7^GMP is a phosphoamide bond (Duclos et al., 1991). Amino acids with nucleophilic side chains including lysine, arginine, asparagine, glutamine, serine, threonine, tyrosine, and cysteine were used to replace His68 (Lin et al., 2012). All the mutants, except H68C, failed to form the covalent [m^7^GMP-enzyme] intermediate. H68C retained a detectable activity for the covalent intermediate formation despite at considerably lower extent. The bond connecting m^7^GMP and the H68C mutant enzyme was moderately stable in 0.1 N HCl and 0.1 N NaOH (Lin et al., 2012), a characteristic of a phosphocysteine bond (Duclos et al., 1991). The change of the nature of the bond connecting the enzyme and m^7^GMP and the result of peptide mapping lead to the conclusion that His68 acts as the nucleophile to attack the α-phosphate of m^7^GTP, consequently leading to the formation of the covalent [m^7^GMP-enzyme] intermediate.

The catalytic step after formation of the [m^7^GMP-enzyme] intermediate was characterized by monitoring the transfer of ^32^P-radiolabeled m^7^GMP of the covalent intermediate to various RNAs (Huang et al., 2005). A RNA transcript with 5′-terminal diphosphate is a prerequisite to receive m^7^GMP from the covalent intermediate, and RNA led by GDP is a better substrate than that led by ADP. The putative stem-loop structure in the 5′ region of BaMV genome, nts 34–118, has a critical effect on the capping efficiency of the genomic RNA, suggesting that most of the cap formation events occur after the stem-loop sequence has been synthesized in nascent transcripts. This result also implies that the RNA polymerase domain and the capping domain of REP_BaMV_ need to coordinate to some extent.

**FIGURE 2:**
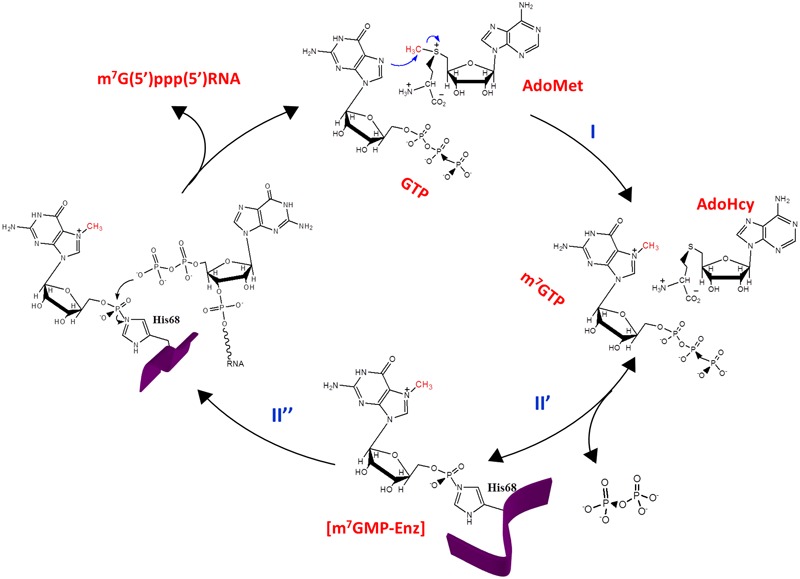
**The cap0 formation pathway of BaMV.** The AdoMet-dependent guanylyltransferase activity exhibited by REP_BaMV_ is composed of activities of (I) GTP methyltransferase and (II) m^7^GTP:RNA guanylyltransferase, which can be further divided into two half reactions with the [m^7^GMP-enzyme] adduct as the intermediate.

According to the data aforementioned and others, the cap formation pathway catalyzed by the capping domain of REP_BaMV_ is delineated in **Figure 2**. (1) GTP and AdoMet bind to the capping domain of REP_BaMV_ in proximity (Hu et al., 2011). The presence of AdoMet actually enhances the binding affinity of the domain for GTP. (2) The precise disposition of GTP and AdoMet in the domain facilitates a nucleophilic attack of the N7 of GTP on the methyl group of AdoMet, leading to the production of m^7^GTP and *S*-adenosyl-L-homocysteine (AdoHcy). (3) The nitrogen atom (not determined whether N^δ1^ or N^𝜀2^) of His68 functions as a nucleophile attacking the α-phosphate of m^7^GTP, under the assistance of Mg^2+^, to form the covalent [m^7^GMP-enzyme] intermediate (Lin et al., 2012). This step is reversible because excess pyrophosphate could drive the m^7^GMP moiety on the covalent intermediate backward to form m^7^GTP (Huang et al., 2004). (4) The 5′-terminal diphosphate of nascent RNA binds to the domain in proximity to the m^7^GMP moiety. The 5′ β-phosphate of the RNA launches a nucleophilic attack on the phosphorus atom of m^7^GMP, leading to the break of the phosphohistidine bond. (5) Finally, the RNA with a 5′ cap0 structure is released from the domain.

## Helicase-Like Domain (HLD)

The HLD of REP_BaMV_ (residues 514–895) forms inclusion bodies when it is expressed in *E. coli*. This domain resumes soluble after denaturation and refolding processes. The purified HLD is able to remove the γ phosphate from nucleoside triphosphates as well as RNA (Li et al., 2001b); in other words, it can be a nucleoside triphosphatase (NTPase) or RNA 5′-triphosphatase (5′-TPase), depending on the substrate. Both of these reactions required the presence of divalent Mg^2+^ or Mn^2+^ cations. Mutations at any of the signature motifs I, II, III, or VI of SF1 abrogate both types of activity (Han et al., 2007). Adenylyl-imidodiphosphate (AMPPNP), a non-hydrolyzable ATP analog, is a competitive inhibitor of the RNA 5′-TPase activity. The inhibition constant *K*i_(AMPPNP)_ was determined to be 93 μM, which is close to the *K*m value of ATP (150 μM) for the NTPase activity (Han et al., 2007). The closeness between the values of *K*i_(AMPPNP)_ and *K*m_(ATP)_ and the simultaneous inactivation of both activities by mutations at the featured motifs of helicases suggest that a common catalytic site is used for the hydrolysis of both NTP and RNA. Nonetheless, the greater value of *K*m_(ATP)_ than *K*m_(RNA)_, which is about 2.5 fold, suggests that more active-site residues are involved in RNA binding. The peptidyl regions employed by the HLD to bind biotinylated RNA were mapped by the reversible formaldehyde crosslinking method followed by tandem mass spectrometry (Han et al., 2009). Five peptidyl regions were identified. Regions of residues 625–645 and 696–706 encompass the helicase motif I and II, respectively; while regions of residues 585–610, 789–799, and 833–843 do not contain conserved sequences known to SF1. Compared with the well-characterized members in SF1, e.g., DNA helicase PcrA, the BaMV HLD seems to bind RNA using a different set of peptidyl regions. Mutagenesis of positively charged residues in these regions showed that some residues, e.g., K603 and R628, have a role in the virus movement (Han et al., 2009).

TGBp1 of BaMV is also a member of SF1. TGBp1 is capable of hydrolyzing NTPs but not RNA (Li et al., 2001b), implying that the RNA 5′-TPase activity embedded in the HLD of REP_BaMV_ is not necessarily a property of all helicase proteins. The biological relevance of the RNA 5′-TPase activity was demonstrated in an *in vitro* assay, in which an RNA transcript would be capped at the 5′ end by the capping domain of REP_BaMV_ only if the RNA transcript had been pretreated with the HLD (Li et al., 2001b). Taken together, the first two domains of REP_BaMV_ work in a concerted manner to complete the formation of the 5′ cap on the nascent viral positive-strand RNAs. Besides participating in 5′ cap formation, the HLD of REP_BaMV_ has also been proposed to act as a *bona fide* helicase in the replication/transcription process of BaMV. Unfortunately, convincing evidence for duplex RNA-unwinding activity is still lacking even though a great deal of time and effort has been spent. To our knowledge, no helicase activity has been reported in the HLD of any other potexviruses. Perhaps, a more sophisticated assay is needed to discern this peculiar helicase activity. It is also possible that a host protein (other than a host helicase) may be recruited as an accessory subunit of the helicase to confer unwinding activity on the viral protein.

Yeast two-hybrid screen using a cDNA library prepared from BaMV-infected leaves of *N. benthamiana* identified a strong protein–protein interaction between the HLD of REP_BaMV_ and the viral CP (Lee et al., 2011). Interacting with CP does not alter the *in vitro* enzymatic activity of the HLD. Mutations of A209G and N210S in CP, which diminish the CP-HLD interaction, were identified by a bacterial two-hybrid screen using a CP random mutant library generated by error-prone PCR (Lee et al., 2011). Mutant BaMV carrying A209G and/or N210S reproduces as efficiently as the wild type virus in *N. benthamiana* protoplasts (Lee et al., 2011). CP with the mutations retains a full activity for RNA binding, and the mutant virions exhibit similar morphologies as the wild type under transmission electron microscope (Lee et al., 2011). Nonetheless, the CP mutations do exert a profound effect on BaMV cell-to-cell movement in plants. With the A209G mutation, BaMV spreads much less effectively in leaves of *N. benthamiana* and *Chenopodium quinoa* (Lee et al., 2011). Notably, A209 of BaMV CP is well conserved among many potexviruses such as PVX and *Foxtail mosaic virus* (FoMV). A230G mutation in FoMV CP, analogous to BaMV A209G, also reduces the viral HLD-CP interaction and restricts the cell-to-cell movement of FoMV in *C. quinoa* (Lee et al., 2011). This finding suggests that the HLD-CP interaction is rather common in potexviruses; moreover, this interaction is relevant to the ability of the virus to move between cells. The critical role of the HLD-CP interaction in BaMV movement prompts us to suspect that REP_BaMV_ is recruited into the viral movement complex, which is composed of mainly the viral RNA, TGBps, and CP. More importantly, REP_BaMV_ may pass through plasmodesmata along with the viral RNA. With this strategy, the viral RNA can be re-replicated immediately in the newly invaded cells so that the virus has a greater chance to defeat the silencing mechanism imposed by the hosts. Involvement of the replication protein in the viral movement complex has also been proposed in TMV based on the observation that TMV requires a significantly longer time for movement from primary inoculated cells to secondary cells than is required for movement from secondary to tertiary cells (Kawakami et al., 2004).

## A Distinct Pathway/Machinery for the 5′ Cap Formation

**FIGURE 3:**
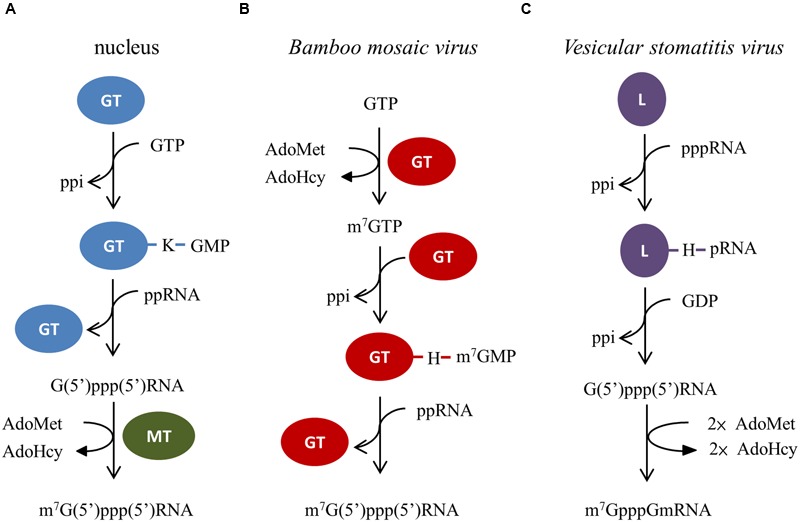
**The distinct cap formation pathways among eukaryotic nucleus, *Bamboo mosaic virus*, and *Vesicular stomatitis virus*. (A)** In nucleus, GTP:mRNA guanylyltransferase (GT) and RNA (guanine-N7) methyltransferase (MT) are responsible for the cap formation. **(B)** Only the capping enzyme domain (GT) of REP_BaMV_ is required for *Bamboo mosaic virus* to form the cap structure. **(C)** Whereas, the L protein of *Vesicular stomatitis virus* possesses both the activities of RNA:GDP polyribonucleotidyltransferase and mRNA methyltransferase.

The 5′ cap0, m^7^G(5′)ppp(5′)Np, in eukaryotic mRNAs is a basic structural unit required for mRNA export from the nucleus, prevention of mRNA degradation by 5′-exonucleases, and recognition by eIF4F complex to initiate the translation process (Furuichi and Shatkin, 2000; Shuman, 2001). Different pathways leading to the formation of the cap structure have been reported (**Figure 3**). Three consecutive enzymatic reactions are responsible for the cap formation in the nucleus. First, the γ-phosphate of a nascent mRNA is removed by RNA 5′-triphosphatase. The GMP moiety of GTP is then transferred to the 5′ end of the 5′-diphosphorylated mRNA via a covalent enzyme-lysyl-GMP intermediate by GTP:mRNA guanylyltransferase. Finally, the guanine-N7 of G(5′)ppp(5′)Np cap is methylated by RNA (guanine-N7) methyltransferase to produce the cap0 structure (Mizumoto and Kaziro, 1987; Shuman, 1995). This canonical cap formation pathway also occurs in some DNA viruses, e.g., vaccinia virus (Shuman et al., 1980; Niles and Christen, 1993) and chlorella virus (Håkansson et al., 1997), and the double-stranded RNA reovirus (Luongo et al., 2000; Luongo, 2002). In the case of BaMV, the RNA 5′-triphosphatase activity embedded in the helicase-like domain of REP_BaMV_ catalyzes the removal of γ-phosphate from the 5′ end of nascent positive-strand RNA (Li et al., 2001b). The capping domain of REP_BaMV_ exhibits an AdoMet-dependent mRNA guanylyltransferase activity, by which the methyl group of AdoMet is transferred to the N7 of GTP, and then the m^7^GMP moiety is transferred from the newly formed m^7^GTP to the 5′ end of a 5′-diphosphorylated RNA via a covalent enzyme-histidyl-m^7^GMP intermediate (Li et al., 2001a,b; Huang et al., 2005; Lin et al., 2012). Plausibly, this type of cap formation pathway for BaMV also occurs across the alphavirus-like superfamily of human, animal, and plant-infection positive-strand RNA viruses. *Vesicular stomatitis virus* (VSV) performs another unconventional mRNA 5′ cap formation pathway (Ogino and Banerjee, 2007; Ogino et al., 2010). Besides exhibiting a RNA-dependent RNA polymerase activity, the L protein of VSV has a RNA:GDP polyribonucleotidyltransferase activity that catalyzes the transfer of the 5′-monophosphorylated viral mRNA to GDP via an enzyme-histidyl-pRNA intermediate. Two methylation reactions at the capped RNA follow to form the cap1 structure by the viral methyltransferase activity.

The BaMV enzymes performing the catalytic steps in the pathway are also unique from the viewpoint of protein structures. Apparently, the BaMV RNA 5′-triphosphatase activity has emerged from the helicase motif-containing domain. By contrast, the RNA 5′-triphosphatases of yeast and DNA viruses, e.g., vaccinia virus and baculovirus, belong to a metal-dependent phosphohydrolase family (Lima et al., 1999), while those of animals and plants are classified into a cysteine phosphatase superfamily (Changela et al., 2001). Moreover, the capping domain of REP_BaMV_ does not share similarity in amino acid sequence with either GTP:mRNA guanylyltransferase or RNA (guanine-N7) methyltransferase of eukaryotic cells and DNA viruses. With the limited genome size, BaMV has evolved an efficient capping enzyme, with merely 442 amino acids, to accomplish the work of forming the 5′ cap.

## RNA-Dependent RNA Polymerase Domain

The C-terminal domain of REP_BaMV_ had been thought to be the key component of the viral replication complex, due to the presence of the hallmark signature of polymerase S/TGX3TX3NS/TX22GDD (Koonin and Dolja, 1993). This domain (residues 893–1364), expressed in *E. coli* with a thioredoxin tag fused at the N terminus, exhibits an *in vitro* RNA polymerase activity, preferentially taking the 3′-terminal fragments of both positive and negative strands of BaMV as templates (Li et al., 1998). Mutational analysis confirmed the essential role of the GDD motif in the catalysis of polymerization reaction. Structure mapping based on selective RNA hydrolysis using a variety of ribonucleases and chemicals suggested that the 3′ UTR of BaMV folds into four consecutive stem-loop domains (A–D), followed by a tertiary pseudoknot structure (Cheng and Tsai, 1999). The hexanucleotide ACC/UUAA, conserved in the 3′ UTR of potexviruses, is situated in the apical loop of the D domain. A competition binding assay suggested that the *E. coli*-expressed BaMV polymerase domain binds independently to the D domain and the poly(A) tail (Huang et al., 2001). A footprinting assay further defined the D loop as the primary region protected by the polymerase domain of REP_BaMV_ against chemical cleavages (Huang et al., 2001). Similarly, the 3′-terminal fragment (77 nts) of the BaMV negative-strand RNA was mapped to contain a 5′stem-loop, followed by a spacer and the 3′-CUUUU sequence (Lin et al., 2005). Reducing the number of uridylate in the 3′-CUUUU to less than three or changing the penultimate U to other nucleotides is deleterious to BaMV accumulation in plants (Chen et al., 2010). UV-crosslinking and competition assay indicated that the *E. coli*-expressed BaMV polymerase domain also binds to the 3′-terminal fragment of the negative-strand RNA through a specific interaction particularly with the 5′stem-loop (Chen et al., 2010). In summary, the polymerase domain of REP_BaMV_ recognizes the specific sequence and structural feature formed on the 3′-terminal region of both the positive and negative strands of BaMV, enabling the viral RNA replication to be initiated at the precise positions. Without these specific protein–RNA interactions, the replication of the viral RNAs would be incorrect or even impossible.

## Subcellular Localization of REP_BaMv_

In general, the replication complexes of plant positive-strand RNA viruses, which consist of the viral replication proteins, the viral genomic RNAs, and co-opted host factors, are embedded in membrane-enclosed micro-compartments derived from various cellular organelles (Novoa et al., 2005; Miller and Krijnse-Locker, 2008). Virus replication within the microenvironments should benefit the viral RNAs from being destroyed by the host defense mechanisms. For instances, *Brome mosaic virus* and *Red clover necrotic mosaic virus* recruit the membrane derived from endoplasmic reticulum to constitute their replication complexes (Noueiry and Ahlquist, 2003; Turner et al., 2004), while *Flock house virus* and *Tomato bushy stunt virus* employ the membrane of mitochondria and peroxisome, respectively, for replication complex assembly (Miller et al., 2001; McCartney et al., 2005). REP_BaMV_ is also a membrane-associated protein. In fact, the membrane fraction P30 of BaMV-infected leaves, the pellet of cell extract after 30000 × *g* centrifugation, exhibits an *in vitro* BaMV RNA-dependent RNA polymerase activity; therefore, the P30 has been used in analysis of the *cis*-acting RNA elements required for the viral genome replication (Chen et al., 2003, 2005, 2010; Lin et al., 2005). To locate the subcellular organelle where BaMV replicates, a genetically modified BaMV positive-strand RNA that contains a phage MS2 CP-recognized sequence was inoculated into *N. benthamiana* leaves that had been infiltrated with *Agrobacterium tumefaciens* carrying the NLS-GFP-MS2 fusion protein-encoding gene (Cheng et al., 2013). The viral RNA was found located in chloroplasts according to the green fluorescent imaging of the infected cells under confocal microscope. Therefore, BaMV was proposed to replicate in chloroplasts although REP_BaMV_ *per se* was invisible in the virus-infected leaves under microscope due to the low expression amount.

The chloroplast is a common target of a large number of plant viruses belonging to a variety of genera. Subcellular localization of the virus-encoded proteins in the chloroplast may constitute a basis for the viral pathogenesis or/and is critical for the viral propagation (Zhao et al., 2016). Besides BaMV, *Alternanthera mosaic virus* (AltMV) and PVX are two other potexviruses that have been demonstrated to be associated with chloroplasts in their infection processes. The TGB3 of AltMV preferentially accumulates around the chloroplast membrane and disruption of TGB3 targeting to chloroplast impairs cell-to-cell movement of the virus (Lim et al., 2010). Furthermore, AltMV TGB3 strongly interacts with the photosystem II oxygen-evolving complex protein PsbO and this interaction correlates with chloroplast vesiculation and veinal necrosis caused by TGB3 over-expression (Jang et al., 2013). In the case of PVX, the viral CP interacts with the transit peptide of plastocyanin, a protein involved in photosynthesis, and silencing of plastocyanin prior to PVX infection reduces CP accumulation in chloroplasts and ameliorates symptom severity in host plants (Qiao et al., 2009).

## Host Proteins Associated with REP_BaMv_

A number of approaches have been used in the search for host proteins involved in regulation of the polymerase activity of REP_BaMV_. A biochemical protocol, basically involving steps of (1) UV-induced crosslinking of proteins in leaf cell extract to the ^32^P-radiolabeled 3′ UTR of BaMV, (2) nuclease digestion, and (3) radiolabeled protein identification using mass spectrometry, has identified several 3′ UTR-interacting proteins including chloroplast phosphoglycerate kinase (PGK), cytosolic glyceraldehyde 3-phosphate dehydrogenase (GAPDH), and heat shock protein 90 homolog (NbHsp90). PGK promotes BaMV accumulation presumably by facilitating transport of the viral genomic RNA to chloroplasts, the plausible replication site for BaMV (Lin et al., 2007; Cheng et al., 2013). GAPDH binds to the pseudoknot poly(A) tail of BaMV and reduces the replication efficiency of the viral negative-strand RNA probably through a competition with REP_BaMV_ for RNA binding (Prasanth et al., 2011). NbHsp90 enhances BaMV replication presumably by either promoting the maturation of REP_BaMV_ or bridging the interaction of REP_BaMV_ with the viral RNA (Huang et al., 2012). The physical interaction between NbHsp90 and REP_BaMV_ was actually confirmed by a yeast two-hybrid assay. Yeast two-hybrid screen was also used to search for host proteins interacting with the polymerase domain of REP_BaMV_. An uncharacterized host AdoMet-dependent methyltransferase (PNbMTS1) was thus isolated from the cDNA library prepared from *N. benthamiana* leaves (Cheng et al., 2009). PNbMTS1 exhibits an AdoMet-dependent inhibitory effect on BaMV CP accumulation in protoplasts. By contrast, *Tobacco rattle virus*-induced gene silencing of PNbMTS1 increased BaMV CP and genomic RNA in *N. benthamiana*. Both the membrane-targeting signal peptide and the AdoMet-binding motifs are essential for PNbMTS1 to suppress BaMV accumulation. Collectively, PNbMTS1 may have a role in the plant innate defense mechanism. Nonetheless, the target of PNbMTS1 relevant to the inhibition effect is still unknown.

Recently, we found that the expression of REP_BaMV_ in *N. benthamiana* could be significantly enhanced if satBaMV was co-expressed. Probably, the positive-strand RNA of satBaMV might act as a template to facilitate the folding of REP_BaMV_ or prevent REP_BaMV_ from being degraded by host proteases. Based on this finding, a proteomic approach was set up to find out the plant proteins differentially present in the REP_BaMV_-enriched P30 fraction (Lee et al., 2016). This approach includes steps of (1) transient expression of the hemagglutinin tag (HA)-fused REP_BaMV_ and satBaMV, or satBaMV alone as the comparative control, in *N. benthamiana* by agroinfiltration, (2) preparation of the P30 fraction from the agroinfiltrated leaves, (3) protein solubilization using anionic detergent Sarkosyl, (4) protein precipitation using anti-HA antiserum, and (5) identification of the co-precipitated proteins by tandem mass spectrometry. Accordingly, dozens of host proteins were identified. To examine the role of the proteins in BaMV replication, each of the genes was transiently silenced in *N. benthamiana*. Those plants without apparent changes in phenotype were then challenged with a genetically modified BaMV that carries *GFP* as a reporter gene. Several potential host factors affecting BaMV replication were thus identified based on the effect of gene silencing on GFP expression. A cytoplasmic 5′→3′ exoribonuclease (NbXRN4), a ripening-related protein, *S*-adenosylmethionine synthetase, and a respiratory burst oxidase homolog were found capable of promoting BaMV replication. By contrast, NADP^+^-dependent isocitrate dehydrogenase and MAP kinase phosphatase-like protein appeared to suppress BaMV replication. The relevance between the activity of NbXRN4 and BaMV replication was further investigated. In brief, NbXRN4 benefits BaMV replication, probably by removal of the uncapped genomic and subgenomic RNAs produced erroneously during the replication/transcription process.

## Perspective

Studies on replication-related proteins of plant RNA viruses have long been limited by inefficient protein expression and difficulty in protein purification. The catalytic characteristics of REP_BaMV_ may thus not only apply to other members of the *Potexvirus* but also serve as references for those of other genera that also belong to the alphavirus-like superfamily. Nonetheless, the structural information at the atomic level regarding the functional domains of REP_BaMV_ is still lacking, thanks mostly to the aggregation nature of these viral proteins. Methods that can overcome this obstacle are urgently needed. The search for host proteins, including those either boost or attenuate the enzymatic activity of REP_BaMV_, should be continued. More importantly, the mechanism underlying the function of host proteins should be elucidated so that the holistic and dynamic interplay between REP_BaMV_ and its host can be understood.

## Author Contributions

MM organized the contents of the text and wrote the manuscript. C-CL participated into the discussion about the text contents and approved the manuscript.

## Conflict of Interest Statement

The authors declare that the research was conducted in the absence of any commercial or financial relationships that could be construed as a potential conflict of interest.
